# A scoring system for predicting the difficulty of trans-radial cerebral angiography

**DOI:** 10.3389/fneur.2026.1762959

**Published:** 2026-07-31

**Authors:** Bing-Sen Xie, Xiao-Rong Shan, Jin-Hong Ma, Shu Zhang, Shufa Zheng, Jing Wang, Xin-Long Wan, De-Zhi Kang

**Affiliations:** 1 Department of Neurosurgery, the First Affiliated Hospital of Fujian Medical University, Fuzhou, China; 2 Department of Neurosurgery, the People’s Hospital of Yuanzhou District, Guyuan, China; 3 Department of Neurology, the People’s Hospital of Yuanzhou District, Guyuan, China; 4 Department of Radiology, the People’s Hospital of Yuanzhou District, Guyuan, China

**Keywords:** aortic arch anatomy, cerebral angiography, predictive, scoring system, trans-radial access

## Abstract

**Background:**

Despite the growing adoption of trans-radial access in neurointervention, no validated scoring system currently exists to predict procedural difficulty of trans-radial cerebral angiography.

**Purpose:**

To develop and validate a scoring system for pre-procedural assessment of trans-radial cerebral angiography difficulty.

**Materials and methods:**

We recruited consecutive patients who underwent trans-radial cerebral angiography between June 2024 and April 2025. Patients were grouped according to the modified operator rating scale based on previous literature and assigned to either the easy or hard group. Baseline characteristics, imaging measurements, procedural information, and complications were recorded. The scoring system was developed through multivariable logistic regressions.

**Results:**

Forty-nine patients were included in this study (mean age, 63.53 ± 1.57 years, 31 men). The easy group included 26 patients, and the hard group included 23 patients. Independent predictors of trans-radial cerebral angiography difficulty were diameter of aortic arch at apex ≥28 mm (OR = 4.651, 95% CI = 1.099–19.658, *p* = 0.037) and total number of vascular angulations (OR = 3.680, 95% CI = 1.441–9.395, *p* = 0.006). A scoring system (range: 0–5 points) was derived, with scores ≥ 3 indicating high difficulty.

**Conclusion:**

Patients with a larger diameter of aortic arch, and more tortuous vessels have a higher difficulty of trans-radial cerebral angiography. This scoring system may be useful for predicting difficulty in pre-procedural trans-radial cerebral angiography.

## Introduction

There is a growing shift from trans-femoral access (TFA) to trans-radial access (TRA) in neurointerventional procedures, driven by demonstrated patient benefits such as fewer serious access-site complications, shorter hospitalization, reduced costs, and higher patient satisfaction (1–7). Nevertheless, TRA can present considerable technical challenges in certain cases, occasionally necessitating conversion to alternative access routes—a scenario that elevates both patient risk and procedural expense (8).

To address this limitation, pre-procedural computed tomography angiography (CTA) of the aortic arch has emerged as a useful preparatory step. It enables detailed anatomical evaluation, helps anticipate variations or difficulties, and supports the advance selection of suitable devices and techniques. Recent studies indicate that specific anatomical features—such as a large aortic arch diameter, a double subclavian-innominate curve, and the absence of bovine arch anatomy—are correlated with higher procedural complexity during trans-radial cerebral angiography (9).

However, pre-procedural aortic arch CTA is not universally performed, and no standardized approach currently exists to translate imaging-derived anatomical factors into a reliable prediction of TRA difficulty. In clinical practice, many patients undergo chest computed tomography (CT) prior to cerebral angiography, yet such scans still capture relevant morphological details of the aortic arch and supra-aortic vessels. The recent research indicates that the advantages of CT imaging include its wide application, high-resolution imaging capability, and the ability to provide comprehensive anatomical details. These characteristics make it a valuable tool for preoperative planning of endovascular treatment (EVT) (10). This study therefore aims to develop a novel CT-based anatomical classification of the aortic arch and supra-aortic branches, identify independent predictors of TRA difficulty, and derive a practical scoring system for pre-procedural assessment in trans-radial cerebral angiography.

## Materials and methods

### Study design

**Figure 1:**
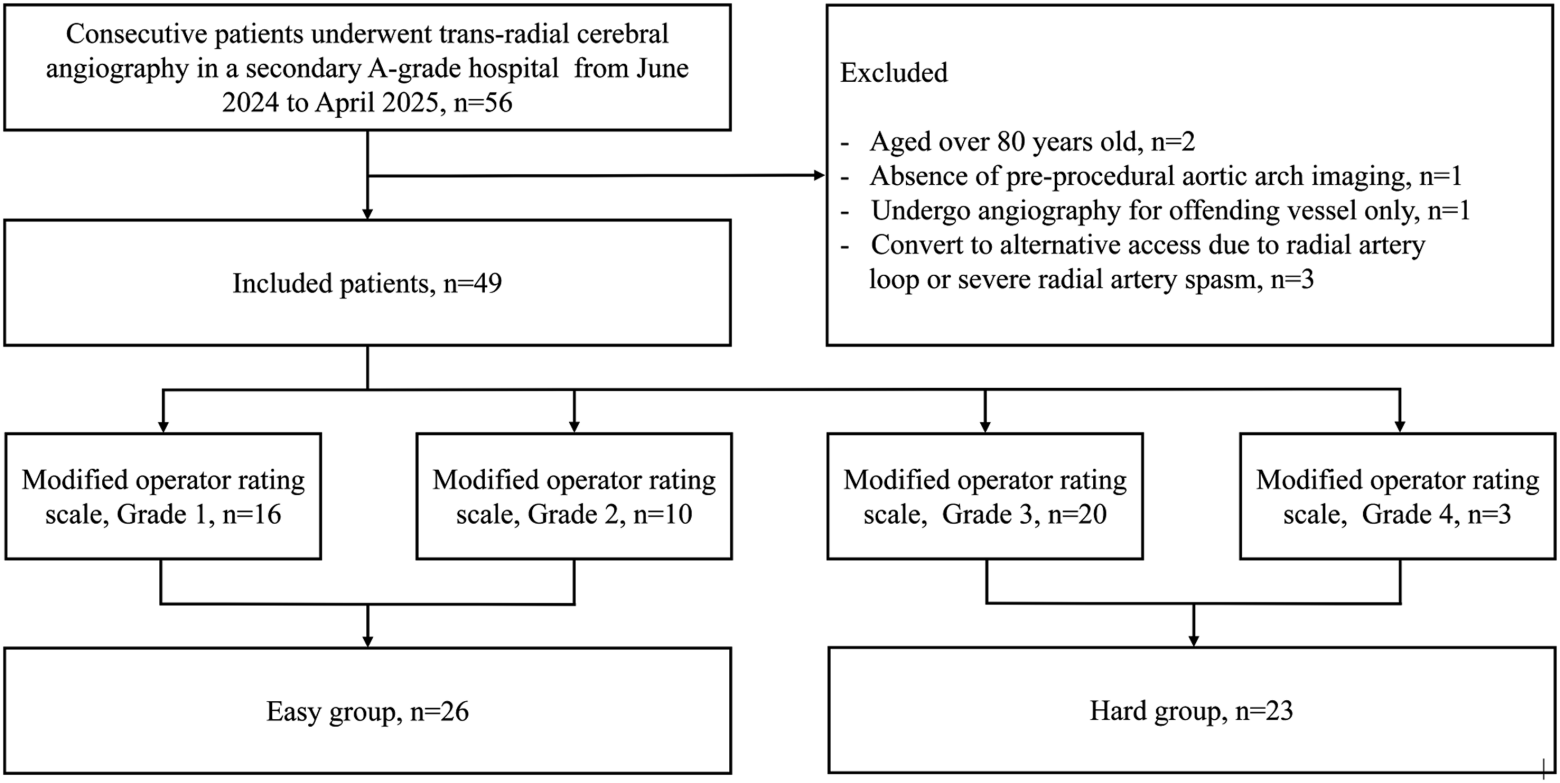
Study flowchart.

The studies involving human participants were reviewed and approved by the People’s Hospital of Yuanzhou District and affiliation of ethics committee. The studies were conducted in accordance with the local legislation and institutional requirements. Written informed consent from the patients was not required to participate in this study in accordance with the national legislation and the institutional requirements. We enrolled consecutive patients who underwent trans-radial cerebral angiography in a secondary A-grade hospital from June 2024 to April 2025. The inclusion criteria were: patients aged 18–80 years, undergoing trans-radial cerebral angiography for the first time. Exclusion criteria included: absence of pre-procedural aortic arch imaging data, angiography performed only for offending vessel to reduce contrast agent dosage, conversion to an alternative access due to radial artery loop or severe radial artery spasm, where such conditions are identified after radial artery sheath insertion but prior to the commencement of cerebral angiography. Patients were stratified according to the modified operator rating scale based on previous literature (9) and assigned to either the easy group (Grade 1 and Grade 2) or hard group (Grade 3 and Grade 4) as follows: Grade 1, no problems accessing vessels of interest, procedure time-per-vessel ≤ quartile (Q) 1 of procedure time-per-vessel of all patients; Grade 2, took longer than usual to catheterize vessels of interest, Q 1 < procedure time-per-vessel ≤ Q 2; Grade 3, required attending to scrub in, able to catheterize vessels of interest with difficulty, procedure time-per-vessel > Q 2; Grade 4, unable to catheterize vessels of interest, converted to another site for access or stop, procedure time-per-vessel > Q 2. Baseline characteristics, imaging measurements, procedural details, and postoperative complications were analyzed and compared between groups. The study flowchart is shown in Figure 1.

### Clinical management

A standardized clinical protocol was used in the management of patients. The operators were neurointerventional fellows with experience of a minimum of 150 prior trans-radial cerebral angiography procedures before participating in this study. TRA is typically performed for cerebral angiography via the right radial artery, using a 5 French Simmons 2 glide catheter (Cordis Corp., Minnesota, United States) and a 0.035-inch Radifocus guidewire (Terumo Corp., Tokyo, Japan). Routine angiography includes a four-vessel study of the bilateral common carotid arteries and subclavian arteries. Selective angiography of the internal carotid artery, external carotid artery, or vertebral artery is performed only when clinically indicated and after confirming the absence of significant stenosis at the origin of the target vessel during the procedure. The following principles were followed during trans-radial cerebral angiography: complete the planned angiography as comprehensively as possible, minimize catheter exchanges, reduce procedural duration, and avoid access conversion unless absolutely necessary.

### Data collection and definition

We collected the following data: patient age, gender, body mass index (BMI), smoking status, alcohol consumption, medical history, pathology, “TRA” grading scale (9), imaging measurements, procedure time, modified operator rating scale, and postoperative complications.

**Figure 2:**
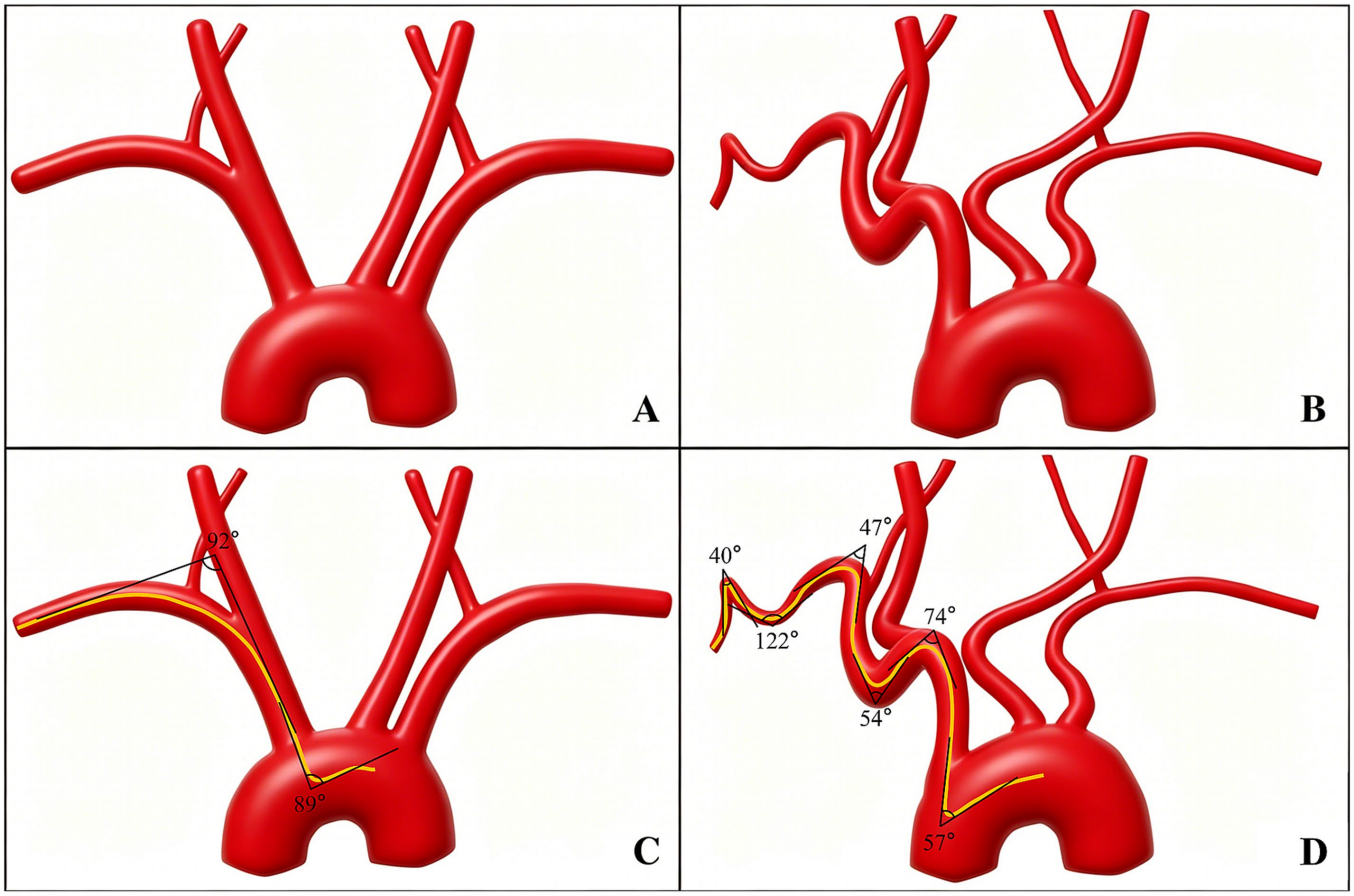
Quantification of vascular tortuosity. **(A)** and **(B)** show normal and tortuous blood vessels, respectively. Along the catheter navigation path from the right axillary artery to the aortic arch, the vascular centerline was delineated [orange curves in **(C,D)**], and the angles along the centerline were measured. **(C)** Exhibits 2 curves, with angles of 92° and 89°, respectively. **(D)** Exhibits 5 curves, with angles of 40°, 47°, 54°, 74°, and 57°, respectively. Notably, the 122° angle was excluded from the tortuosity count as it exceeds the 120° threshold.

Imaging measurements from aortic arch CTA and/or chest CT were performed by two independent fellows. In cases of disagreement, a third fellow reviewed the data to reach consensus. First, we analyzed chest CT scans of enrolled patients to evaluate aortic arch and supra-aortic branch anatomy. Findings were compared with aortic arch CTA and digital subtraction angiography (DSA) to assess chest CT’s capability in characterizing aortic arch and supra-aortic branch anatomy. Based on existing literature and empirical observations, we translated vascular features affecting trans-radial cerebral angiography difficulty into quantifiable CT parameters, establishing an imaging anatomic classification system. The primary challenges in trans-radial cerebral angiography involve catheter navigation and shaping (11–13). Thus, the CT-based imaging anatomic classification evaluates three dimensions: vascular variations, aortic arch morphology and vascular tortuosity. Vascular tortuosity was quantified based on the catheter pathway drawn from previous literature (14). Based on the course of the right axillary artery to the aortic arch, a centerline was plotted from the proximal axillary artery to the left common carotid artery ostium. The angulations along this centerline were measured and categorized into mild (>60°), moderate (30–60°) or severe (<30°) based on the previous described classification systems (15, 16). Since the catheter tends to straighten the vessel during advancement, angles >120° were not considered tortuous. Both magnitude and number of angulations were recorded (Figure 2).

Procedure time-per-vessel was defined as the interval between completion of previous angiography and initiation of current angiography. For the first vessel, this represented the duration from radial artery angiography completion to first vessel angiography commencement.

Major complications included hematoma, radial artery vasospasm, radial artery occlusion, compartment syndrome, and perioperative stroke, all receiving appropriate treatment.

### Statistical analysis

Statistical analyses were conducted using R, version 4.4.1 (R Foundation for Statistical Computing, Vienna, Austria). Data were presented as mean ± standard deviation (SD), median (range), and percentages. Continuous variables were analyzed using independent samples *t*-tests, categorical variables with *χ*^2^ or Fisher’s exact tests. The multivariate logistic regression model with backward elimination was used to identify independent predictors of trans-radial cerebral angiography difficulty. Variables showing *p* ≤ 0.20 in univariate analysis were entered as potential predictors. Model predictive ability was evaluated using receiver operating characteristic (ROC) curve analysis, calculating the area under the curve (AUC). For internal validation, 1,000 bootstrap samples were generated, and bootstrap-corrected AUCs and calibration plots were produced. We computed adjusted odds ratios (ORs) with 95% confidence intervals (CIs). A scoring system was developed based on multivariable logistic regression coefficients. Statistical significance was set at *p* < 0.05.

## Results

### Patient population

During the study period, 56 consecutive patients underwent first-time trans-radial cerebral angiography at our institution. Exclusions comprised two patients over the age of 80 years, one without pre-procedural aortic arch imaging, one undergoing targeted angiography for a single suspected offending vessel, and three requiring alternative access due to radial artery loop or severe spasm. Thus, 49 patients comprised the final cohort (Figure 1).

### Patient characteristics

Baseline characteristics are summarized in Table 1. The cohort comprised 49 patients (63.3% male; mean age 63.53 ± 1.57 years). Patients were stratified into easy (*n* = 26) and hard (*n* = 23) groups, with no significant intergroup differences in baseline characteristics (*p* > 0.05).

**Table 1:** Baseline characteristics of patients.

Characteristics	Total (*n* = 49)	Easy group (*n* = 26)	Hard group (*n* = 23)	*p* value
Age, year				
Mean ± SD	63.53 ± 1.57	62.46 ± 2.20	64.74 ± 2.27	0.476
Range	34–79	34–79	37–79	
Gender, *N* (%)				0.130
Male	31 (63.3)	19 (73.1)	12 (52.2)	
Female	18 (36.7)	7 (26.9)	11 (47.8)	
BMI, kg/m^2^	25.32 ± 0.43	25.88 ± 0.62	24.69 ± 0.58	0.170
Smoking, *N* (%)	12 (24.5)	9 (34.6)	3 (13.0)	0.080
Drinking, *N* (%)	3 (6.1)	2 (7.7)	1 (4.3)	0.999
Medical history, *N* (%)				
Hypertension	35 (71.4)	16 (61.5)	19 (82.6)	0.103
Diabetes mellitus	12 (24.5)	5 (19.2)	7 (30.4)	0.363
Hyperlipidemia	11 (22.4)	6 (23.1)	5 (21.7)	0.911
Pathology, *N* (%)				0.879
Artery stenosis	24 (49.0)	13 (50.0)	11 (47.8)	
Others	25 (51.0)	13 (50.0)	12 (52.2)	
“TRA” grading scale, point				
Mean ± SD	3.02 ± 0.33	2.88 ± 0.49	3.17 ± 0.43	0.662
Range	0–8	0–8	0–7	
Procedure time-per-vessel, min				
Mean ± SD	5.25 ± 0.35	3.86 ± 0.12	6.82 ± 0.59	—
Range	2.50–16.25	2.50–4.60	4.75–16.25	
Q 1	4.00	3.48	5.00	
Q 2	4.60	4.00	6.00	
Q 3	5.90	4.31	7.25	
Modified operator rating scale, median (range)	2 (1–4)	1 (1–2)	3 (3–4)	—

SD, standard deviation; *N*, number; BMI, body mass index; Q, quartile.

### Capability of chest CT

Of the 49 patients, 47 (95.92%) received preoperative aortic arch CTA and 17 (34.69%) underwent chest CT. The right vertebral artery was not clearly visualized in 3 cases and the left vertebral artery in 2 cases in chest CT, whereas other major arteries were clearly identifiable.

### CT-based imaging anatomic classification

The CT-based imaging anatomic classification is showed in Table 2. Regarding vascular variations, no patients exhibited an aberrant right subclavian artery (ARSA), while a bovine aortic arch was observed in 2 patients, both of whom were in the easy group. For aortic arch morphology, there were 6 (12.2%) cases of type 2 and 4 (8.2%) cases of type 3 aortic arch. The hard group (7 cases, 30.4%) had slightly more patients with type 2/3 arch compared to the easy group (3 cases, 11.5%), though this difference was not statistically significant (*p* = 0.200). The mean aortic arch diameter at the apex measured 29.38 ± 0.50 mm, with the hard group (30.38 ± 0.70 mm) being marginally larger than the easy group (28.50 ± 0.67 mm), but again without statistical significance (*p* = 0.059). When the aortic arch diameter at the apex was dichotomized as “≥28 mm” and “<28 mm” (9), the hard group (18 cases, 73.3%) included more patients with diameters ≥28 mm compared to the easy group (13 cases, 50.0%), showing a statistically significant difference (*p* = 0.041). In vascular tortuosity assessment,the median number of angles was 0 for 30–60° and 2 for angles exceeding 60°, yielding a median total vascular angulations of 2. Notably, the hard group showed statistically significant increases in both the number of angles >60° and the total vascular angulations compared to the easy group.

**Table 2:** Imaging anatomic classification for the aortic arch and supra-aortic branches of patients.

Variables	Total (*n* = 49)	Easy group (*n* = 26)	Hard group (*n* = 23)	*P* value
Variation, *N* (%)				
Presence of ARSA	0 (0)	0 (0)	0 (0)	—
Presence of bovine arch	2 (4.1)	2 (7.7)	0 (0)	0.526
Aortic arch morphology, *N* (%)				
Type of aortic arch				0.200
Type 1	39 (79.6)	23 (88.5)	16 (69.6)	
Type 2	6 (12.2)	1 (3.8)	5 (21.7)	
Type 3	4 (8.2)	2 (7.7)	2 (8.7)	
Diameter of aortic arch at apex				
Mean ± SD, mm	29.38 ± 0.50	28.50 ± 0.67	30.38 ± 0.70	0.059
≥ 28 mm, *N* (%)	31 (63.3)	13 (50.0)	18 (73.3)	0.041
Tortuosity, median (range)				
<30°	0 (0–0)	0 (0–0)	0 (0–0)	—
30–60°	0 (0–2)	0 (0–2)	0 (0–1)	0.305
>60°	2 (0–5)	2 (0–4)	3 (1–5)	0.002
Total number of vascular angulations	2 (1–5)	2 (1–4)	3 (2–5)	0.003

*N*, number; ARSA, aberrant right subclavian artery; SD, standard deviation.

### Postoperative complications

Careful monitoring during follow-up revealed no evidence of postoperative complications.

### Independent predictors of trans-radial cerebral angiography difficulty

All baseline and imaging anatomic variables (gender, BMI, smoking status, hypertension, aortic arch type, arch diameter at the apex, and total number of vascular angulations) with *p* ≤ 0.20 in univariate analysis were included in the multivariate logistic regression model. To simplify the model, BMI was categorized into: normal weight (18.5–25.0 kg/m^2^), overweight (25.1–29.9 kg/m^2^), and obese (>30 kg/m^2^) (17, 18). Aortic arch diameter was dichotomized as <28 mm and ≥28 mm^9^. Due to collinearity between total number of vascular angulations and number of angles >60°, only the former was retained in multivariate analysis. After adjustment, aortic arch diameter ≥28 mm (OR = 4.651; 95% CI = 1.099–19.658; *p* = 0.037) and total number of vascular angulations (OR = 3.680; 95% CI = 1.441–9.395; *p* = 0.006) remained independent predictors of difficulty in trans-radial cerebral angiography (Table 3). The ROC curve for this model showed an AUC of 0.839 (95% CI = 0.722–0.955), and the bootstrap corrected AUC was 0.858 (95% CI = 0.747–0.953).

**Table 3:** Multivariate analysis for predictors of the difficulty trans-radial cerebral angiography.

Predictors	Unadjusted		Adjusted	
	OR (95% CI)	*P* value	OR (95% CI)	*P v*alue
Diameter of aortic arch at apex ≥ 28 mm	3.600 (1.027–12.617)	0.045	4.651 (1.099–19.685)	0.037
Total number of vascular angulations	3.141 (1.400–7.048)	0.006	3.680 (1.441–9.395)	0.006

OR, odd ratio; CI, confidence interval.

### A scoring system for predicting the difficulty of trans-radial cerebral angiography

**Figure 3:**
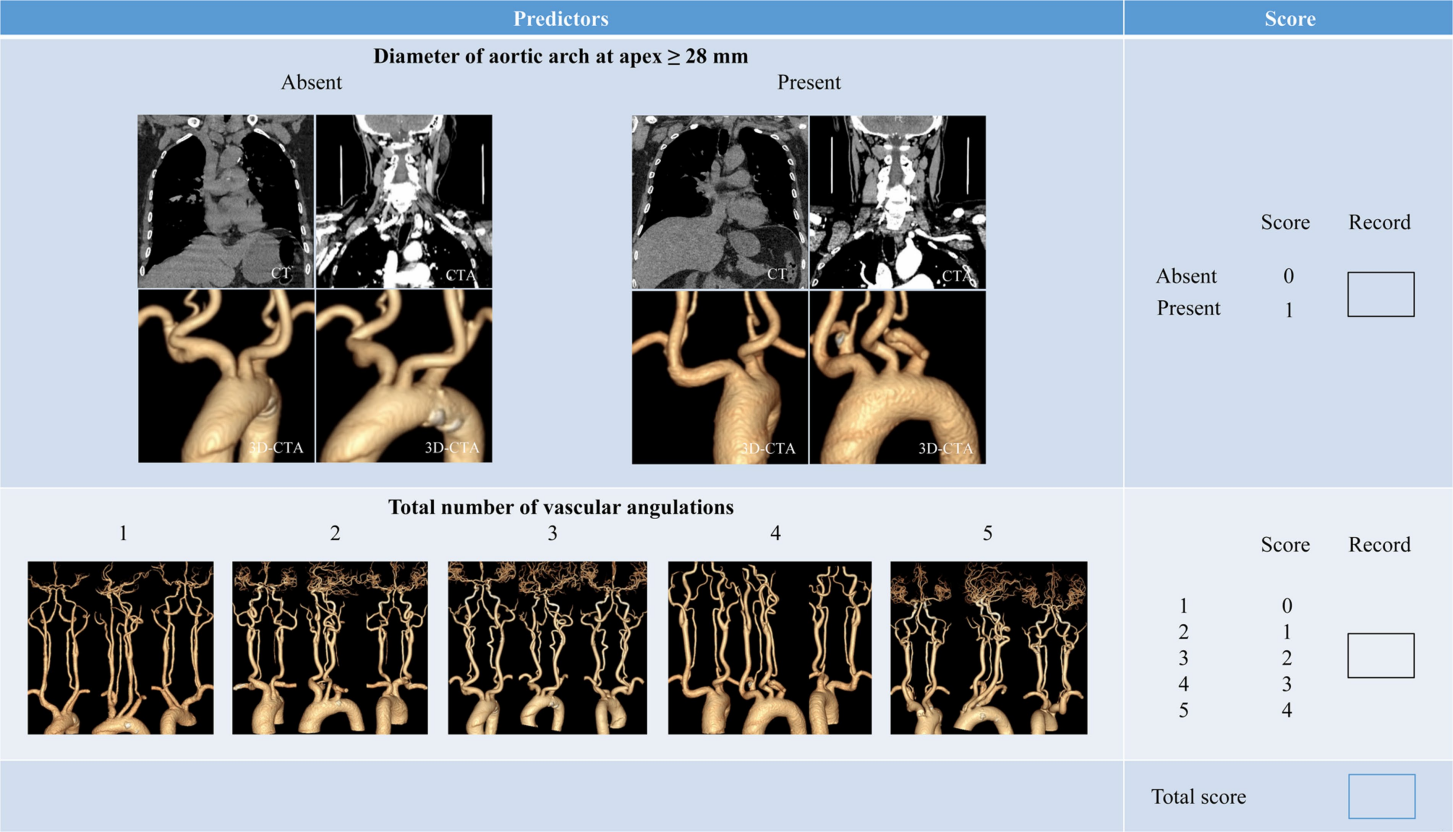
Scoring system for predicting the difficulty of trans-radial cerebral angiography. The left column shows representative images for each sign. Enter scores in the black record box and display the total score in the blue box below.

Based on the multivariate logistic regression coefficients, a clinical scoring system was developed. The regression coefficients were proportionally converted to integer scores, using the smallest coefficient (*β* = 1.303) as the reference for 1 point. The assigned scores were as follows: 1, 2, 3, 4, and 5 angulations received 0, 1, 2, 3, and 4 points, respectively; aortic arch diameter <28 mm received 0 points, and ≥28 mm received 1 point. The total score ranged from 0 to 5 points. The optimal cut-off value, determined by the Youden index, was 3 points, yielding a sensitivity of 56.5% and a specificity of 88.5%. Accordingly, patients were stratified into a low-risk group (score 0–2) and a high-risk group (score 3–5) (Figure 3).

### “TRA” grading scale

**Figure 4:**
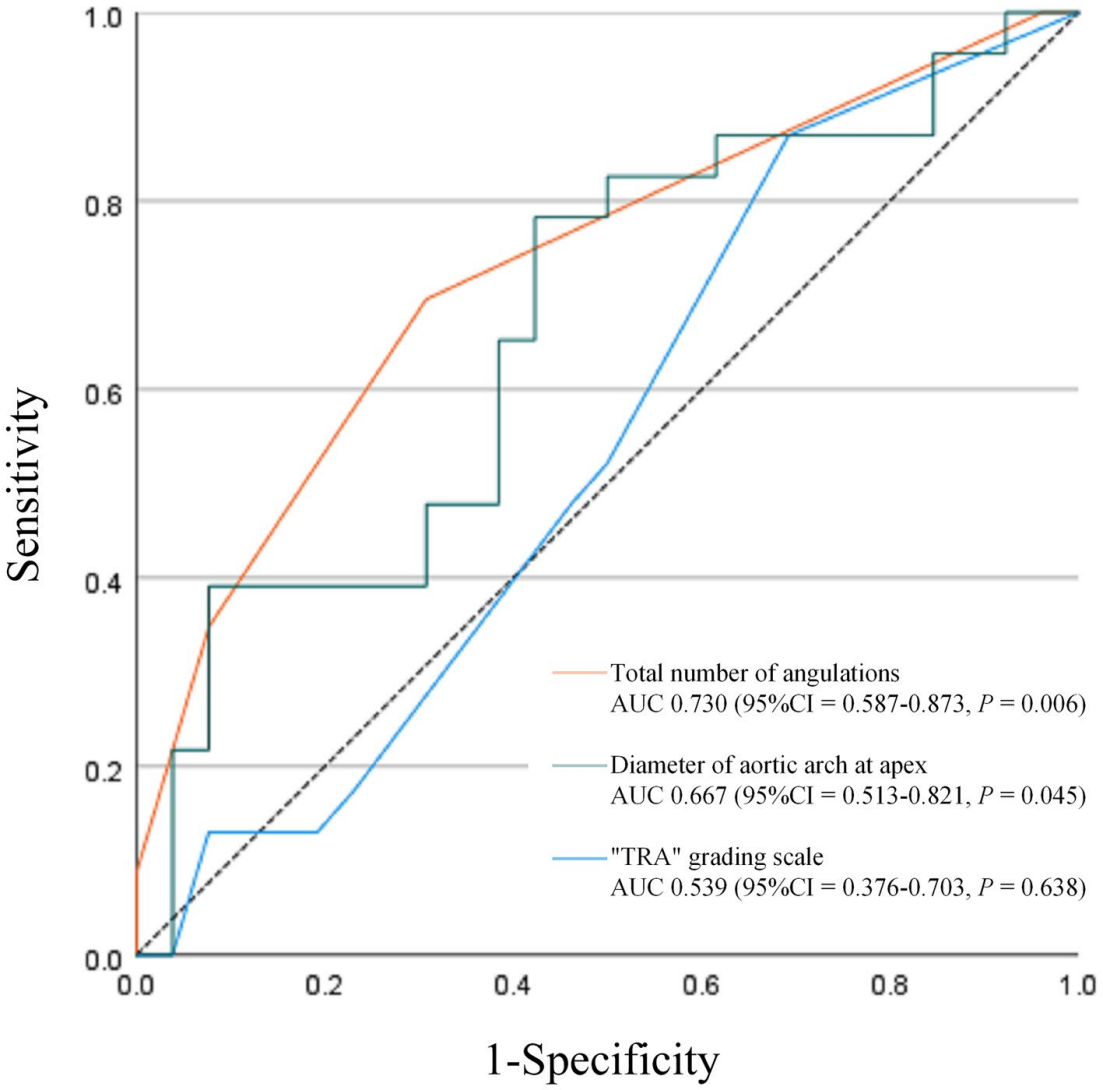
ROC curve analysis of the TRA score, the presence of an aortic arch diameter ≥28 mm at the apex, and the total number of angulations in trans-radial cerebral angiography difficulty. The AUC of the “TRA” grading scale was 0.539 (95% CI = 0.376–0.703, *p* = 0.638), the AUC of the total number of angulations was 0.730 (95% CI = 0.587–0.873, *p* = 0.006), and the AUC of the aortic arch diameter at the apex was 0.667 (95% CI = 0.513–0.821, *p* = 0.045). ROC, receiver operating characteristic; AUC, area under the curve; CI, confidence interval.

The “TRA” grading scale of patients in the hard group (3.17 ± 0.43) was higher than that of patients in the easy group (2.88 ± 0.49), though without a significant difference (*p* = 0.662). The “TRA” grading scale was positively correlated with the type of aortic arch, while the diameter of the aortic arch at the apex and the total number of angulations were not correlated (Table 4). The difficulty of trans-radial cerebral angiography was predicted by comparing the TRA score, the presence of an aortic arch diameter ≥28 mm at the apex, and the total number of angulations using ROC curves. The AUC of the “TRA” grading scale was 0.539 (95% CI = 0.376–0.703, *p* = 0.638), the AUC of the total number of angulations was 0.730 (95% CI = 0.587–0.873, *p* = 0.006), and the AUC of the aortic arch diameter at the apex was 0.667 (95% CI = 0.513–0.821, *p* = 0.045) (Figure 4).

**Table 4:** Results of Spearman correlation between “TRA” grading scale and the anatomical factors of aortic arch and supra-aortic branches.

Variables	Correlation coefficients	*P* value
Type of aortic arch	0.443	0.001
Diameter of aortic arch at apex	0.263	0.067
Total number of vascular angulations	0.084	0.567

## Discussion

**Figure 5:**
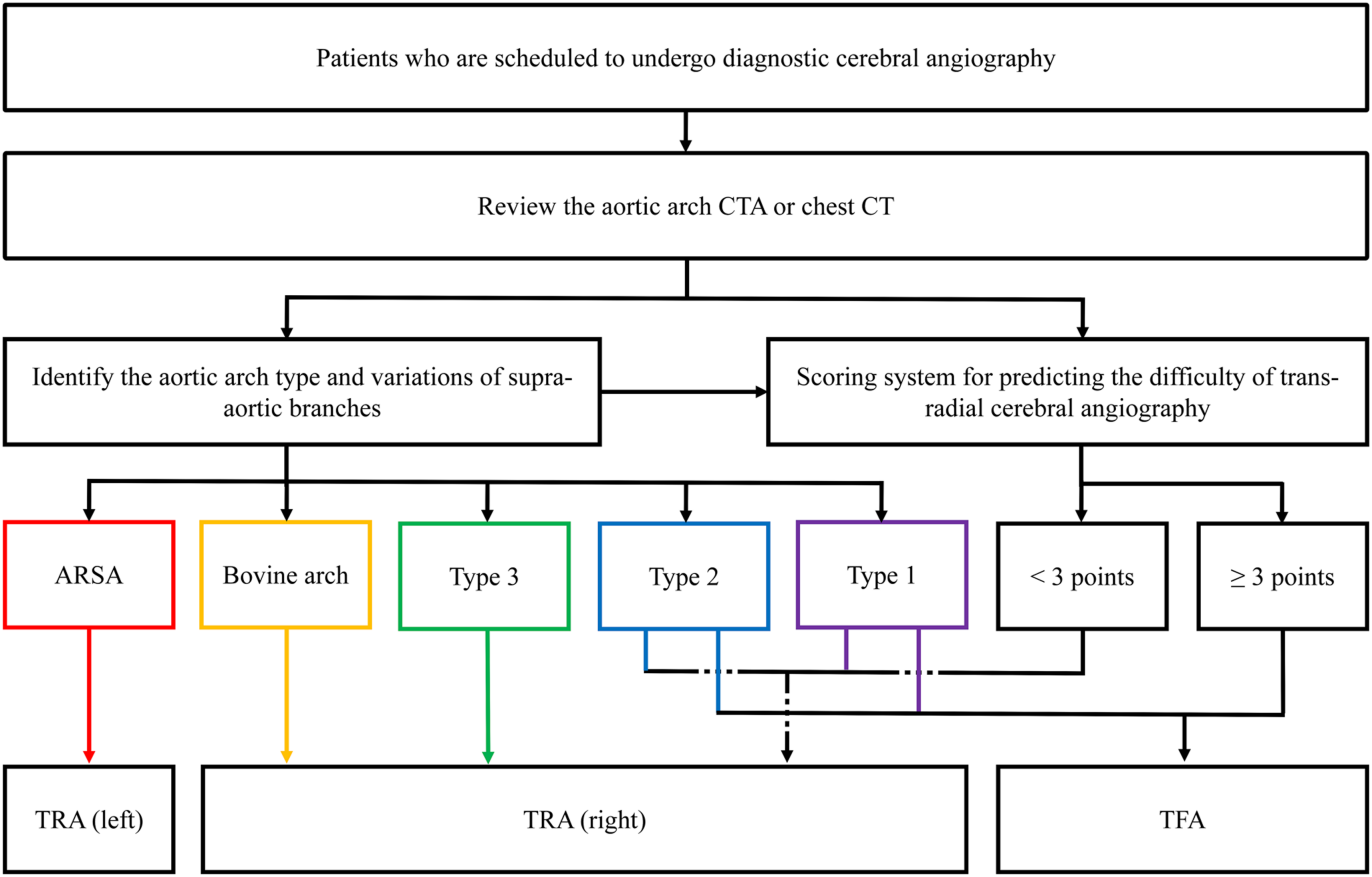
A standardized preoperative assessment protocol to evaluate procedural trans-radial cerebral angiography difficulty and access selection. CTA, computed tomography angiography; CT, computed tomography; ARSA, aberrant right subclavian artery; TRA, trans-radial access; TFA, trans-femoral access.

In this study, we established a CT-based imaging anatomical classification system using aortic arch CTA or chest CT imaging. Univariate and multivariate analyses of baseline characteristics and this classification scheme identified two independent predictors of procedural difficulty in trans-radial cerebral angiography: the diameter of the aortic arch at apex and the total number of vascular angulations. These parameters were used to develop a clinical scoring system. Compared with previously published models (9, 19–22), our prediction model is more parsimonious and practical, facilitating direct and rapid application in clinical practice despite the single-center retrospective design and modest sample size of this study (Table 5). In addition, we developed a standardized preoperative assessment protocol for evaluating procedural difficulty, which may assist in optimal selection of interventional access (Figure 5).

**Table 5:** Comprehensive comparison of literature on predicting difficulty of trans-radial cerebral angiography.

Criterion	Khan et al. (9)	Choi et al. (19)	Eun et al. (20)	Hu et al. (21)	Tian et al. (22)	This study
Journa Study Design Primary Focus Key Anatomical Parameters Key Findings (OR/*β*) Prediction Model Strengths Limitations	J NeuroInterv Surg Retrospective analysis (*n* = 52) To identify anatomical and clinical factors that make trans-radial cerebral angiography more difficult, and to introduce a novel grading scale for predicting procedural difficulty • Aortic arch diameter (>28 mm) • Double subclavian-innominate curve • Left CCA proximal loop • Acute subclavian-vertebral angle • Proximal radial loop • Bovine arch (protective) Aortic arch diameter (*p* < 0.01); double subclavian-innominate curve (*p* < 0.01); left CCA loop (*p* < 0.001); acute subclavian-vertebral angle (*p* < 0.01); absence of bovine arch (*p* = 0.03) “TRA Grading Scale” First systematic study to identify anatomical risk factors for TRA difficulty; pioneering grading scale Small sample size; grading scale not prospectively validated	J NeuroInterv Surg Retrospective analysis (*n* = 263) To investigate the anatomical predictors of the aortic arch and its branches that impede navigation of the left ICA in right TRA • Aortic arch type (Casserly I–III) • Height of right subclavian artery (cutoff 54.83 mm) • Left CCA turnoff angle (cutoff 17°) • Innominate-to-left CCA distance (cutoff 4.25 mm) • Left CCA diameter (cutoff 6.05 mm) • Vessel angulation grade (tortuous/kinking/looping) Left CCA turnoff angle OR = 0.953 per 1° (*p* = 0.0017); innominate-to-left CCA distance OR = 1.784 (*p* < 0.0001); right subclavian height OR = 1.071 (*p* = 0.0068); type III arch OR = 6.323 (*p* = 0.0171) Multivariable logistic regression model (AUC = 0.969) Relatively large sample size; clinically applicable cutoff values; excluded bovine arch to reduce confounding Focused exclusively on left ICA navigation; excluded complex cases (radial loops, bovine arch); single-center	J NeuroInterv Surg Retrospective analysis (*n* = 306) To predict the success of left ICA selective angiography using preprocedural CT and MR imaging of aortic arch factors • A1: Upward angle of subclavian artery • A2: Right subclavian-innominate angle • A3: Innominate-left CCA angle (key) • D1: Aorta-to-right subclavian length • D2: Innominate-to-left CCA length (key) A3 OR = 0.95 per 1° (*p* < 0.001); D2 OR = 1.19 per 1 mm (*p* = 0.001) Online prediction tool (AUC = 0.87) Largest sample size; interactive online prediction tool Failure group only 42 cases (13.7%), class imbalance; single-center; only Simmons 2 catheter evaluated	Clin Neurol Neurosurg Retrospective analysis (*n* = 210) To explore anatomical and clinical factors affecting radiographic exposure time in TRA cerebral angiography and to establish a neural network prediction model • Aortic arch diameter • Innominate artery width and length • Innominate-aortic arch angle • Left CCA-aortic arch angle • Left subclavian-aortic arch angle • Double subclavian-innominate curve • Left CCA proximal loop Aortic arch diameter *β* = 0.256 (*p* < 0.001); innominate width *β* = 0.317 (*p* < 0.001); innominate-aortic angle *β* = 0.140 (*p* = 0.042); innominate length *β* = −0.156 (*p* = 0.024); hypertension (*p* = 0.014); double tortuosity (*p* < 0.001); left CCA loop (*p* = 0.014); age (*p* = 0.002) Neural network model (accuracy: 80.0% training / 76.9% testing; AUC = 0.821) First to apply neural network; identified novel parameters (innominate width/length); robust internal validation (bootstrap AUC = 0.858) Neural network lacks interpretability; single-center; dichotomization by median may lose information	BMC Neurol Retrospective analysis(*n* = 170) To investigate the impact of arterial tortuosity (particularly dual subclavian-brachiocephalic tortuosity) on procedural efficiency in TRA cerebral angiography and elucidate the mechanistic pathway • Radial artery tortuosity • Brachial-axillary artery curve • Subclavian-brachiocephalic anatomy (normal/single curve/isolated brachiocephalic curvature/dual tortuosity) • Aortic arch type • Bovine arch Dual tortuosity OR = 4.70 (*p* = 0.001); age OR = 1.05 (*p* = 0.001); BMI OR = 1.14 (*p* = 0.005); smoking OR = 1.96 (*p* = 0.037) Ordinal logistic regression + parallel mediation analysis Mediation analysis demonstrated that 95.5–99.2% of the effect of dual tortuosity on fluoroscopy time is mediated by left vessel catheterization; mechanistic clarity Single-center, retrospective; qualitative anatomical assessment (not quantitative); excluded radial access failures	— Retrospective analysis (*n* = 49) To develop a novel CT-based anatomical classification, identify independent predictors of TRA difficulty, and derive a practical scoring system for pre-procedural assessment in trans-radial cerebral angiography • Aortic arch diameter • vascular angulation Diameter of aortic arch at apex ≥28 mm OR = 4.651 (*p* = 0.037); Total number of vascular angulations OR = 3.680 (*p* = 0.006) Multivariate logistic regression model (AUC = 0.839) First to developed a standardized preoperative assessment protocol; parsimonious prediction model; robust internal validation (bootstrap AUC = 0.858) Single-center; retrospective; small sample size; no external validation

ICA, internal carotid artery; TRA, trans-femoral access; CCA, common carotid artery; OR, odd ratio; AUC, area under the curve.

The preferred access for all patients scheduled for diagnostic cerebral angiography is TRA. Alternative access routes should be considered only when pre-procedural assessment indicates significantly increased technical difficulty with TRA and the risk–benefit ratio becomes unfavorable. The first step involves identifying the aortic arch type and variations of supra-aortic branches, including Type 1, Type 2, and Type 3 arches, bovine arch, and ARSA. Although no ARSA cases were encountered in our study, literature suggests that such patients may undergo cerebral angiography via left radial access (9, 23–25). The typical morphology in patients with Type 3 aortic arch or bovine arch applies for an arterial access from the right upper extremity (26). For patients with Type 1 or Type 2 aortic arches whose scoring system result exceeds 3 points, trans-radial cerebral angiography may present greater difficulty with potentially longer procedure times and increased radiation exposure. To minimize procedural duration, we recommend TFA for these cases.

According to literature reports, the presence of a double subclavian-innominate curve is associated with greater difficulty in TRA cases (9, 21, 22). In our study, the double subclavian-innominate curve introduces two additional angulations, corresponding to a 1-point increase in our scoring system. Consequently, the total score invariably exceeds 3 points, which indicates significantly increased procedural difficulty for trans-radial cerebral angiography. Tortuosity may also exist along the axillary-to-subclavian arterial segment—a factor frequently overlooked in previous studies. Among our patients with 4–5 angulations, most exhibited tortuosity in this specific segment, thereby increasing the total angulation count. Therefore, we propose quantifying vascular tortuosity by counting angulations along the entire axillary artery-to-aortic arch pathway. This method may proves both simpler and more clinically practical compared with alternative approaches reported in the literature (27–29).

The “TRA” grading scale ranges from 0 to 8 points without significant intergroup differences in our case series, indicating the scale’s limited predictive value for trans-radial cerebral angiography difficulty. This discrepancy may partly stem from two methodological considerations: First, the absence of radial artery condition evaluation in all cases potentially underestimated total scores by 0–2 points. Although incorporating radial artery assessment might improve accuracy, its overall impact would likely remain marginal. Second, the scale disproportionately weights (0–6 points) the vertebral-subclavian angle’s acuity—a parameter of questionable clinical relevance since selective vertebral artery catheterization proves unnecessary during routine bilateral vertebral angiography. Furthermore, our anatomical analysis reveals that while the “TRA” grading scale correlates with aortic arch type, it shows no significant association with established predictors like aortic arch diameter or total number of angulations.

## Limitations

This study has several inherent limitations that should be acknowledged. First, as a retrospective clinical study with a small sample size, the generalizability of findings may be constrained. Second, critical exclusion criteria for TRA—including minimal radial artery diameter and arterial loops—were not incorporated into our scoring system due to unavailable data. However, radial artery loops are rare and can be overcome using advanced catheterization techniques (9, 30–32). Third, we acknowledge that the potential confounding effect of the operator learning curve was not fully eliminated. Although all participating operators had each completed at least 150 transradial cerebral angiography procedures prior to this study, we did not formally assess the inter-rater reliability of our procedural difficulty scoring system. This may have introduced unmeasured inter-operator variability.

## Conclusion

The aortic arch diamete and the total number of vascular angulations have been identified as independent risk factors for trans-radial cerebral angiography difficulty. Based on these findings, we have developed a novel scoring system and corresponding assessment protocol for predicting difficulty in diagnostic cerebral angiography. Multicenter prospective studies with larger sample sizes and standardized reliability assessments are warranted to validate our findings.

## Data Availability

The raw data supporting the conclusions of this article will be made available by the authors, without undue reservation.

## References

[1] Atallah E El Naamani K Momin AA Abbas R Jain P Hunt A . Transradial versus transfemoral access routes for diagnostic cerebral angiography: a large single-center comparative cost-analysis study. J Neurosurg. (2024) 140:1328–34. doi: 10.3171/2023.9.JNS23941, 37976514

[2] Mortezaei A Hajikarimloo B Eraghi MM Sheikholeslami S Sameer O Shahidi R . Trans-radial vs. trans-femoral approaches in diagnostic cerebral angiography: a comprehensive systematic review and meta-analysis of practicality and cost-effectiveness. Clin Neurol Neurosurg. (2024) 247:108637. doi: 10.1016/j.clineuro.2024.108637, 39531956

[3] Catapano JS Fredrickson VL Fujii T Cole TS Koester SW Baranoski JF . Complications of femoral versus radial access in neuroendovascular procedures with propensity adjustment. J Neurointerv Surg. (2020) 12:611–5. doi: 10.1136/neurintsurg-2019-015569, 31843764

[4] Shi M He S. Transradial and transfemoral accesses for cerebral angiography: a retrospective comparative study. Neurol Res. (2023) 45:1063–8. doi: 10.1080/01616412.2023.2257410, 37751776

[5] Stone JG Zussman BM Tonetti DA Brown M Desai SM Gross BA . Transradial versus transfemoral approaches for diagnostic cerebral angiography: a prospective, single-center, non-inferiority comparative effectiveness study. J Neurointerv Surg. (2020) 12:993–8. doi: 10.1136/neurintsurg-2019-015642, 31974282

[6] Batista S Oliveira LB Diniz JBC Pinheiro AC Maia H Duarte M . Transradial versus transfemoral approach in cerebral angiography: a meta-analysis. Interv Neuroradiol. (2023) 32:871–80. doi: 10.1177/15910199231212520, 37936392 PMC13294543

[7] Xia J Xiao S Xv G Li T Wang Z. Effects and safety of the transradial artery approach for cerebral angiography: a comparative observational study. Eur J Radiol. (2025) 188:112165. doi: 10.1016/j.ejrad.2025.112165, 40359733

[8] Ruzsa Z Nemes B Pinter L Berta B Toth K Teleki B . A randomised comparison of transradial and transfemoral approach for carotid artery stenting: RADCAR (RADial access for CARotid artery stenting) study. EuroIntervention. (2014) 10:381–91. doi: 10.4244/EIJV10I3A64, 25042266

[9] Khan NR Peterson J Dornbos ID Nguyen V Goyal N Torabi R . Predicting the degree of difficulty of the trans-radial approach in cerebral angiography. J Neurointerv Surg. (2021) 13:552–8. doi: 10.1136/neurintsurg-2020-016448, 32792364

[10] Sano A Sugimoto T Iwasaki T Miki T Takai S Wakana N . Pre-distance assessment from radial artery to lower extremity arterial lesion. Int J Cardiovasc Imaging. (2025) 41:467–75. doi: 10.1007/s10554-025-03328-7, 39779617 PMC11880034

[11] Patel T Shah S Pancholy S Deora S Prajapati K Coppola J . Working through challenges of subclavian, innominate, and aortic arch regions during transradial approach. Catheter Cardiovasc Interv. (2014) 84:224–35. doi: 10.1002/ccd.25418, 24510527

[12] Dehghani P Mohammad A Bajaj R Hong T Suen CM Sharieff W . Mechanism and predictors of failed transradial approach for percutaneous coronary interventions. JACC Cardiovasc Interv. (2009) 2:1057–64. doi: 10.1016/j.jcin.2009.07.014, 19926044

[13] Valsecchi O Vassileva A Musumeci G Rossini R Tespili M Guagliumi G . Failure of transradial approach during coronary interventions: anatomic considerations. Catheter Cardiovasc Interv. (2006) 67:870–8. doi: 10.1002/ccd.20732, 16649233

[14] Mokin M Waqas M Chin F Rai H Senko J Sparks A . Semi-automated measurement of vascular tortuosity and its implications for mechanical thrombectomy performance. Neuroradiology. (2021) 63:381–9. doi: 10.1007/s00234-020-02525-6, 32816090 PMC7880861

[15] Togay-Isikay C Kim J Betterman K Andrews C Meads D Tesh P . Carotid artery tortuosity, kinking, coiling: stroke risk factor, marker, or curiosity? Acta Neurol Belg. (2005) 105:68–72. 16076059

[16] Weibel J Fields WS. Tortuosity, coiling, and kinking of the internal carotid artery. I. Etiology and radiographic anatomy. Neurology. (1965) 15:7–18. doi: 10.1212/wnl.15.1.7, 14257832

[17] Roy JM Mina S Kaul A Hage S Patil S Musmar B . The impact of access site on procedure time and post-anesthesia care unit (PACU) time in patients undergoing outpatient diagnostic angiograms: a propensity-score matched analysis stratified by body mass index. Clin Neurol Neurosurg. (2025) 248:108660. doi: 10.1016/j.clineuro.2024.108660, 39591886

[18] Roy JM Ahmed MT El Naamani K Saadat N Gaskins W Nguyen A . Procedural outcomes of the transradial versus transfemoral approach for diagnostic cerebral angiograms according to BMI: a propensity score-matched analysis. J Neurosurg. (2025) 142:977–83. doi: 10.3171/2024.7.JNS241183, 39504551

[19] Choi SW Kim S Kim H Kim SR Park IS. Anatomical predictors of difficult left internal carotid artery navigation in transradial access for neurointervention. J Neurosurg. (2023) 139:157–64. doi: 10.3171/2022.9.JNS221642, 36334297

[20] Eun J Song SY Im SH Park HK. Transradial cerebral angiography: predicting left ICA selective angiography success using pre-diagnostic aortic arch factors. J NeuroInterv Surg. (2025) 18:508–12. doi: 10.1136/jnis-2024-022842, 40015955

[21] Hu HH Zhang J Xie SY Hu H Xie S Zhou H . Research on predicting radiographic exposure time in imaging based on neural network prediction models. Eur J Radiol. (2025) 249:108736. doi: 10.1016/j.clineuro.2025.108736, 39874733

[22] Tian Q Liu ZB Zhao YN Gu JC. Dual subclavian-brachiocephalic artery tortuosity and its impact on procedural efficiency in trans-radial access cerebral angiography: a retrospective cohort study. BMC Neurol. (2026) 26:255. doi: 10.1186/s12883-026-04799-4, 41803800 PMC13085326

[23] Barros G Bass DI Osbun JW Chen SH Brunet MC Peterson EC . Left transradial access for cerebral angiography. J Neurointerv Surg. (2020) 12:427–30. doi: 10.1136/neurintsurg-2019-015386, 31649205

[24] Majmundar N Patel P Gadhiya A Patel NV Gupta G Agarwalla PK . Left distal radial access in patients with arteria lusoria: insights for cerebral angiography and interventions. J Neurointerv Surg. (2020) 12:1231–4. doi: 10.1136/neurintsurg-2020-016199, 32546634

[25] Choi Y Chung SB Kim MS. Prevalence and anatomy of aberrant right subclavian artery evaluated by computed tomographic angiography at a single institution in Korea. J Korean Neurosurg Soc. (2019) 62:175–82. doi: 10.3340/jkns.2018.0048, 30840972 PMC6411572

[26] Dahm JB van Buuren F Hansen C Becker J Wolpers HG. The concept of an anatomy related individual arterial access: lowering technical and clinical complications with transradial access in bovine and type-III aortic arch carotid artery stenting. Vasa. (2011) 40:468–73. doi: 10.1024/0301-1526/a000150, 22090180

[27] Wang D Basman C Mahani S Kodra A Pirelli L Mehla P . A novel computed tomographic angiography tortuosity index to predict successful sentinel cerebral embolic protection delivery for Transcatheter aortic valve replacement. Struct Heart. (2022) 6:100021. doi: 10.1016/j.shj.2022.100021, 37273736 PMC10236830

[28] Dougherty G Varro J. A quantitative index for the measurement of the tortuosity of blood vessels. Med Eng Phys. (2000) 22:567–74. doi: 10.1016/s1350-4533(00)00074-6, 11182581

[29] Nishikata M Hirashima Y Tomita T Futatsuya R Horie Y Endo S. Measurement of basilar artery bending and elongation by magnetic resonance cerebral angiography: relationship to age, sex and vertebral artery dominance. Arch Gerontol Geriatr. (2004) 38:251–9. doi: 10.1016/j.archger.2003.10.006, 15066311

[30] Deora S Shah S Patel T. Balloon-assisted tracking in dealing with radial artery loop by transradial approach: a technical report. J Invasive Cardiol. (2014) 26:E61–2. 24791729

[31] Barbeau GR. Radial loop and extreme vessel tortuosity in the transradial approach: advantage of hydrophilic-coated guidewires and catheters. Catheter Cardiovasc Interv. (2003) 59:442–50. doi: 10.1002/ccd.10586, 12891603

[32] Cao W Hu R Tang SZ Jia D Zhu D Qi D . Safety profile of trans-radial approach (TRA) in patients with radial artery loop in neurointervention: retrospective analysis of a TRA-dedicated neurovascular center experience over 4 years. J Clin Neurosci. (2025) 139:111400. doi: 10.1016/j.jocn.2025.111400, 40580758

